# Food as a drug

**DOI:** 10.18632/oncoscience.250

**Published:** 2015-09-23

**Authors:** Paul A. Spagnuolo, Michael A. Rogers

**Affiliations:** School of Pharmacy, University of Waterloo, Kitchener, Ontario, Canada

**Keywords:** nutraceuticals, cancer, leukemia, drug discovery

In his Pulitzer Prize winning novel “The Emperor of all Maladies”, Dr. Siddhartha Mukherjee eloquently summarizes the history of rational drug design and its origins in understanding the impact of a food-derived bioactive compound (i.e., folic acid) in disease pathophysiology. Dr. Mukherjee reminds us that Sidney Farber's 1948 seminal paper[1] in the New England Journal of Medicine set the framework for rational drug design. After observing that administration of folic acid in children with acute lymphoblastic leukemia (ALL) accelerated their disease, Farber and colleagues (notably Subbarao) developed anti-folate compounds to treat ALL. These drugs, aminopterin and later methotrexate, which is still used to this day as a cornerstone of several chemotherapy regimens, revolutionized cancer chemotherapy. In the 1980s, studies revealed that supplementation with all-trans retinoic acid (ATRA), a vitamin A derivative, forced differentiation of HL60 cells in culture. Later it was provided to a female patient with acute promyelocytic leukemia (APML) and she miraculously responded. She is alive and well today[2] and ATRA remains a cornerstone of APML therapy.

These are incredible success stories that have revolutionized the way cancer is treated. Unfortunately, there has been a general failure to systemically evaluate food-derived bioactive compounds (i.e., nutraceuticals) as potential cancer treatments. This failure can partly be attributed to the state of the nutraceutical industry. As a billion dollar and rapidly expanding industry, relaxed regulations make new product formulation and health claims around these often untested products a regular occurrence. Moreover, the industry is marketing-centric (i.e., marketing departments vastly outnumber research and development departments) driven by hype and public perceptions and not necessarily credible science. Given the latter, it is easy to understand why most of the medical and scientific community may ignore these molecules. Oncologists will not (and rightly should not) recommend nutraceutical consumption if the science is not credible. Nonetheless, we are at a cross-road. Frequent consumption of these bioactive compounds by both the general public and the cancer patient is necessitating the medical community to act. Hence, new approaches are direly needed to assess the clinical relevance of nutraceuticals.

A nutraceutical library conducive for high-throughput screening has been developed and implemented to address the aforementioned and limitations listed below. Using a screening approach, our group published a paper focused on a small pilot library of 30 nutraceuticals [3]; however, an ongoing study has evaluated 300 compounds and our current library now stands at over 800. There are numerous, well-designed studies that have provided a framework for this research (summarized in[4]); however, the majority of studies on food-derived molecules have significant limitations that curtail the translatability to clinical successes. As an example, few studies rigorously identify the cell and molecular mechanisms for their activity (i.e., determine the nutraceuticals’ cell or molecular target), identify the actual bioactive (i.e., extracts with unknown composition are typically used as opposed to purified compounds), or include pre-clinical data (i.e., focus on a single cell line with no *in vivo* data). These limitations of nutraceutical studies force a negative perception; however, they can provide a basis for more comprehensive exploration. Mechanistic studies, routed in understanding the structure-function mechanisms will engage the medical community, which is often dismayed by the unsubstantiated health claims.

Our recent work evaluated a commercially available library of natural health products (NHP) and determined that avocatin B, a lipid derived from avocados, induced cell death in acute myeloid leukemia cells [5]. Avocatin B was found to be a potent (IC_50_: 1.5 μM) anti-AML compound that imparted its activity by accumulating in mitochondria via CPT1 (i.e., knockdown of CPT1 or mitochondria resulted in avocatin B-resistant cells) and inhibiting fatty acid oxidation, a pathway that AML cells depend on for survival. The inhibition of fatty acid metabolism resulted in reductions of NADH, NADPH and glutathione leading to ROS-mediated apoptosis. Interestingly, of the 800 compounds assessed in the NHP library only a select few were food-derived (nutraceuticals are a subclass of the NHP family). Since our work is primarily interested in nutraceuticals, it may be argued that it was a very serendipitous finding that the most active compound of the NHP screen was a nutraceutical. Alternatively, it may be that nutraceuticals are typically under analyzed in the context of their therapeutic potential so the most active ones remain to be discovered. Regardless, nutraceuticals are a relatively untapped resource for drug discovery.
